# Signaling Through Nucleic Acid Sensors and Their Roles in Inflammatory Diseases

**DOI:** 10.3389/fimmu.2020.625833

**Published:** 2021-01-28

**Authors:** Haruna Okude, Daisuke Ori, Taro Kawai

**Affiliations:** Laboratory of Molecular Immunobiology, Division of Biological Science, Graduate School of Science and Technology, Nara Institute of Science and Technology (NAIST), Ikoma, Japan

**Keywords:** Toll-like receptor, RIG-I-like receptor, cGAMP synthase, nucleic acid sensing, autoimmune disease, autoinflammatory disease

## Abstract

Recognition of pathogen-derived nucleic acids by pattern-recognition receptors (PRRs) is essential for eliciting antiviral immune responses by inducing the production of type I interferons (IFNs) and proinflammatory cytokines. Such responses are a prerequisite for mounting innate and pathogen-specific adaptive immune responses. However, host cells also use nucleic acids as carriers of genetic information, and the aberrant recognition of self-nucleic acids by PRRs is associated with the onset of autoimmune or autoinflammatory diseases. In this review, we describe the mechanisms of nucleic acid sensing by PRRs, including Toll-like receptors, RIG-I-like receptors, and DNA sensor molecules, and their signaling pathways as well as the disorders caused by uncontrolled or unnecessary activation of these PRRs.

## Introduction

The innate immune system is not only the first line of host defense against invading pathogens but is also essential for the biological responses of the host against various harmful stimuli. Furthermore, its activation subsequently contributes to the activation of the adaptive immune system, which eliminates pathogens to restore host homeostasis. The initiation of immune responses occurs *via* germline-encoded pattern-recognition receptors (PRRs), which recognize widely conserved features in pathogens, termed pathogen-associated molecular patterns (PAMPs), as well as “danger signals”, host components released in response to cell or tissue injury, termed damage-associated molecular patterns (DAMPs). PRRs include Toll-like receptors (TLRs), RIG-I-like receptors (RLRs), Nod-like receptors (NLRs), C-type lectin receptors (CLRs) and the cytosolic DNA sensor proteins. When PRRs are activated, they activate their corresponding downstream signaling cascades leading to the induction of innate immune and inflammatory responses through the production of proinflammatory cytokines, type I interferons (IFNs), and other key molecules such as major histocompatibility (MHC) proteins and costimulatory molecules by macrophages, dendritic cells (DCs), neutrophils, and other nonprofessional immune cells (1). Although PRRs are indispensable for host defense to combat invading pathogens and maintain homeostasis, consequential inflammation by aberrant PRRs signaling is likely to be harmful to the organism.

Among a wide variety of PAMPs, nucleic acids derived from pathogens are recognized by TLRs, RLRs, and cytosolic DNA sensors, which provoke antiviral and inflammatory responses mediated by type I IFNs and proinflammatory cytokines, respectively. However, nucleic acids derived from host cells are also recognized by PRRs under certain conditions, which contributes to autoimmunity and autoinflammation (2). Indeed, accumulating evidence suggests that the excessive activation or dysregulation of nucleic acid-sensing systems is responsible for the pathogenesis of many autoimmune and autoinflammatory diseases and cancers. This review focuses on nucleic acid-sensing receptors, their corresponding ligands, downstream signaling pathways, discrimination between self- and non-self-derived nucleic acids, and related diseases.

## Nucleic Acid-Sensing TLRs

**Figure 1:**
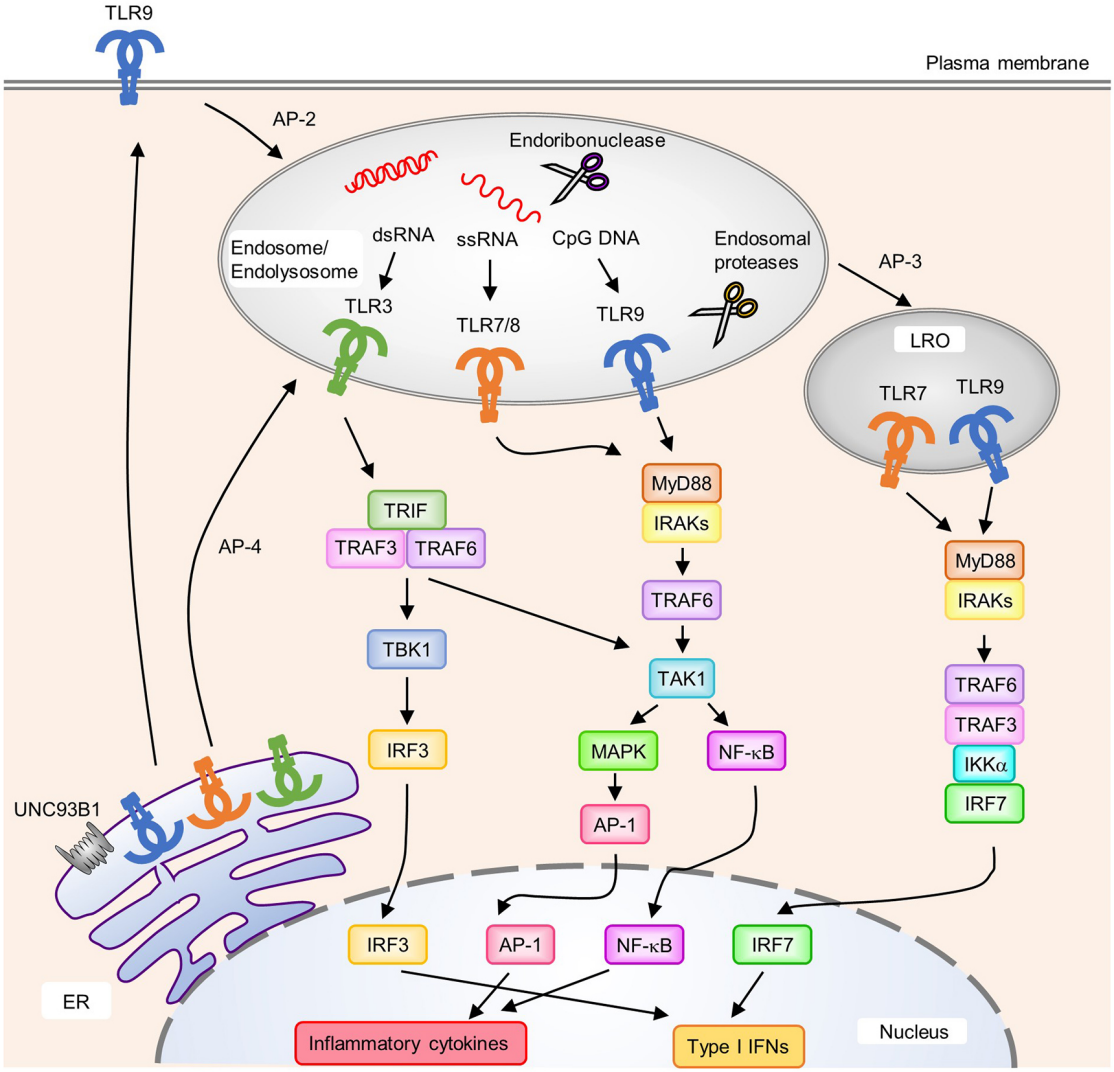
Localization, intracellular trafficking, and signaling pathways of nucleic acid-sensing Toll-like receptors (TLRs). TLR3, TLR7, TLR8, and TLR9 are synthesized in the endoplasmic reticulum (ER) and transported to endosomes *via* UNC93B1. Each TLR is transported to its destination [endosomes, endolysosomes, and lysosome-related organelles (LRO)] by individual mechanisms. TLR9 requires the AP-2 complex to translocate from the cell surface to endosomes, whereas TLR7 interacts with the AP-4 complex for direct trafficking to endosomes. Several endosomal proteases and endoribonucleases in endosomes/endolysosomes process TLRs and nucleic acids, respectively. Upon the recognition of cognate ligands, TLR7, TLR8, and TLR9 recruit MyD88 to activate downstream signaling pathways. MyD88 recruits IRAKs and TRAF6, which subsequently activate TAK1. Activated TAK1 leads to the activation of AP-1 through MAPK to initiate the transcription of proinflammatory cytokines. NF-κB is also activated by TAK1 and induces the production of proinflammatory cytokines. In pDCs, TLR7 and TLR9 in LRO induce the activation of IRF7 by forming a complex with TRAF6, TRAF3, IKKα; and IRF7, which results in the expression of type I IFNs. An AP-3 complex is required for the localization of TLR7 and TLR9 to LRO. TLR3 recruits TRIF to initiate downstream signaling pathway. TRIF recruits TRAF3 and TRAF6 to activate TBK1 and TAK1. Activated TBK1 induces type I IFNs through IRF3, and TAK1 induces proinflammatory cytokine production through NF-κB and AP-1.

The TLR family recognizes a wide variety of PAMPs, ranging from lipids and lipoproteins to nucleic acids derived from microbial pathogens. Among TLRs, TLR3, TLR7, TLR8, TLR9, and TLR13 are predominantly localized to endosomes and recognize nucleic acids (**Figure 1**). TLR3 recognizes double-stranded (ds) RNA, TLR7 and TLR8 recognize single-stranded (ss) RNA, TLR9 recognizes unmethylated CpG DNA, and murine TLR13 recognizes bacterial 23S rRNA to activate downstream signaling pathways that induce inflammatory responses (3–6). Their localization in intracellular compartments is essential for proper ligand recognition, discrimination between self- and non-self-derived nucleic acids, and the activation of downstream signaling pathways. All these TLRs are synthesized in the endoplasmic reticulum (ER), transported to the Golgi apparatus, and eventually recruited to intracellular compartments such as endosomes; however, mechanisms related to their transport from the ER to endosomes varies between individual TLRs. TLR9 requires the adaptor protein-2 (AP-2) complex to mediate its endocytosis from the cell surface to endosomes, whereas TLR7 interacts with the AP-4 complex to mediate direct trafficking from the trans-Golgi network to endosomes (7). For TLR3, the TRIM3-mediated K63-linked polyubiquitination of TLR3 is required for its trafficking from the Golgi apparatus to endosomes by endosomal sorting complex required for transport (ESCRT) complexes (8). Furthermore, lysosomal trafficking regulator (LYST), which mediates phagosomal maturation, was reported to be important for activation of the TLR3 signaling pathway (9).

Compartmentalization into endosomes is important for the recognition of nucleic acids released from phagocytosed pathogens by TLRs while avoiding contact with self-derived nucleic acids (10). UNC93B1, a 12-transmembrane protein in the ER, is a key molecule that interacts with and transports TLRs from the ER to endosomes (**Figure 1**) (7, 11). Consistently, the loss-of-function of Unc93b1 disrupted the TLR3, TLR7, and TLR9 signaling pathways (12). Moreover, endosomal TLR protein levels were reduced in mice harboring a Unc93b1 loss-of-function mutant that impaired its interaction with TLRs, suggesting a role of UNC93B1 in the stabilization of TLR proteins (13). Furthermore, TLR7 and TLR9 are oppositely regulated—TLR9 is predominantly maintained at a steady state, suppressing TLR7 responsiveness and avoiding TLR7-induced autoinflammatory diseases. This predominance of TLR9 was inhibited by a D34A mutation in Unc93B1, which also exacerbated TLR7 activation and systemic lethal inflammation in mice (14). Recently, Unc93b1 was shown to prevent TLR9 activation in intracellular compartments other than endosomes (15). TLR9 is released from Unc93b1 in endosomes, and this disassociation is required for the activation of signaling pathways. However, TLR7 continues to interact with Unc93b1 in endosomes and can activate signaling pathways without dissociation from Unc93b1. The association of Unc93b1 with TLR7 in endosomes is important for terminating TLR7 signaling. Syntenin-1, a PDZ domain-containing adaptor protein, facilitated sorting of the TLR7-Unc93b1 complex from endosomes into the intralumenal vesicles of multivesicular bodies to terminate receptor signaling (16). After stimulation with TLR7, but not TLR9 or TLR3, the interaction of Syntenin-1 with Unc93b1 is increased. Disruption of its binding to Unc93b1 prevents the sorting of TLR7 into multivesicular bodies and results in exaggerated TLR7 signaling. These findings suggest that UNC93B1 regulates the activities of individual endosomal TLRs *via* different mechanisms. The importance of UNC93B1 in human pathology was also demonstrated in patients with a mutation in *UNC93B1* who developed herpes simplex virus (HSV) encephalitis (17). The pathogenesis of HSV encephalitis in UNC93B1-deficient patients is likely caused by impaired TLR3 signaling in neurons and oligodendrocytes (18). The ectodomains of endosomal TLRs undergo proteolytic processing within endosomal compartments by cathepsins and asparagine endopeptidases to generate functional receptors (**Figure 1**) (19, 20). This proteolytic processing is thought to protect against unwanted interactions with self-derived nucleic acids. Indeed, mice expressing TLR9 mutants that accessed the cell surface, and did not require proteolysis for activation, developed systemic and lethal inflammation (21). The pH of intracellular vesicles might also be important for proteolytic processing and ligand recognition of endosomal TLRs. Blockade of the acidification of intracellular vesicles resulted in impaired innate immune responses mediated by TLR3, TLR7, and TLR9 (22, 23). The localization of nucleic acid-sensing TLRs in cellular compartments is also important for the initiation of cell-type-specific signaling pathways. In plasmacytoid DCs (pDCs), a subset of DCs that produce large amounts of type I IFNs *via* TLR7 and TLR9 signaling, the activation of NF-κB mediated by TLR7 or TLR9 was initiated in endosomes, whereas activation of IRF7 for type I IFN expression requires further transport from endosomes to lysosome-related organelles (LRO) *via* an adaptor protein-3 (AP-3)-dependent mechanism (**Figure 1**) (24).

Upon ligand binding, TLRs form a dimer that promotes the association of their intracellular TIR domains, resulting in the recruitment of TIR-containing adaptor proteins such as MyD88 and TRIF (**Figure 1**) (25). Upon ligand recognition, TLR7 and TLR9 recruit MyD88, IRAKs, and TRAF6. IRAKs and TRAF6 complexes subsequently activate TAK1, leading to the activation of NF-κB and mitogen-activated protein kinases (MAPKs). In pDCs, IRAKs and TRAF6 induce the activation of IRF7 by forming a complex that contains IRAK1, TRAF6, TRAF3, IKKα; and IRF7 (26–30). IRAK1 and IKKα phosphorylate IRF7, leading to the translocation of IRF7 into the nucleus (27, 28). In contrast, TLR3 is the only TLR that signals independently of MyD88 by recruiting TRIF upon ligand binding. TRIF interacts with TRAF3 and TRAF6, which promote the activation of TANK-binding kinase 1 (TBK1) and TAK1, respectively. Subsequently, activated TBK1 phosphorylates the pLxIS motif in TRIF, which in turn recruits and activates the transcription factor IRF3 (31). Finally, activated TAK1 activates NF-κB and MAPKs.

## Expression and Ligands of Nucleic Acid-Sensing TLRs

TLR3 is mainly expressed by DCs, fibroblasts, and intestinal epithelial cells (32–34). TLR3 forms a homodimer and binds to 40–50 bp dsRNA, and multiple dimers bind to long dsRNA (35, 36). Although dsRNA longer than 90 bp can bind to TLR3 in early endosomes (pH 6.0–6.5), dsRNA of 40–50 bp is required to form a complex with TLR3 in late endosomes (lower than pH 5.5) (35). Thus, activation of the TLR3-mediated signaling cascade is thought to be dependent on the length of dsRNA and the localization of TLR3. Furthermore, TLR3 is involved in immune responses to several RNA viruses, such as West Nile virus (WNV), Semliki Forest virus, and encephalomyelitis virus (EMCV). DNA viruses such as mouse cytomegalovirus (MCMV) and HSV-1 also elicit TLR3-mediated immune responses, presumably by recognizing dsRNA intermediates from viruses (2). Accordingly, TLR3 is important for protection against HSV-1 infection of the central nervous system (37–39).

Human TLR7 and TLR8, and mouse TLR7, recognize ssRNA from viruses and bacteria, and imidazoquinoline derivatives, such as imiquimod (R837) and resiquimod (R848) (5, 40–42). TLR8 in mice does not respond to ssRNA ligands because of the absence of five amino acids corresponding to amino acids in human TLR8 that are required for RNA recognition (43). Compared with other immune cells, pDCs and B cells predominantly express TLR7 (44, 45). In contrast, TLR8 is strongly expressed in immune cells other than pDC, such as monocytes/macrophages and myeloid DCs (46). Structural analysis revealed that TLR7 and TLR8 have two ligand binding sites in their ectodomains through which TLR7 binds to free guanosine molecules and ssRNAs, and TLR8 binds to free uridine molecules and ssRNAs (47, 48). In the presence of ssRNAs, the affinity of these free nucleotides was enhanced and the binding of both ssRNAs and free nucleotides was important for the efficient activation of TLR7 and TLR8. Because the presence of free nucleotides is required for their activation, the degradation of ssRNAs in lysosomes may be important for ssRNA recognition by TLR7 and TLR8. Indeed, activation of the endolysosomal endoribonucleases RNase T2 and RNase 2 is required for the recognition of pathogen-derived RNA by TLR8 (49, 50). In addition to guanosine and uridine, deoxyguanosine and deoxyuridine can also activate TLR7- and TLR8-induced signaling pathways, respectively in the presence of ssRNA (51). Therefore, together with ssRNA, DNA degradation products also synergistically increase the activation of TLR7 and TLR8, and abnormalities in DNA metabolism may trigger the inflammatory response due to increased activation of TLR7 and TLR8, as well as TLR9. Physiologically, TLR7 and TLR8 are involved in host responses against a variety of RNA viruses, including influenza A virus (IAV), human immunodeficiency virus (HIV), and vesicular stomatitis virus (VSV) (2). Although TLR7 and TLR8 are often considered to be similar, a recent report showed that TLR7 and TLR8 in human monocytes elicited different immune responses (52). In that study, activation of TLR7 promoted the expression of cytokines that induced Th17 cell polarization whereas activation of TLR8 induced the expression of Th1-type cytokines and type I IFNs.

TLR9, mainly expressed by pDCs, B cells, and monocytes/macrophages, recognizes DNA with an unmethylated CpG motif from bacteria and viruses (4). TLR9 forms a complex with CpG DNA at a 2:2 ratio (53). This interaction is increased under acidic conditions, and thus localization to lysosomes may allow TLR9 to recognize DNA. In contrast to CpG DNA, TLR9 has a different binding site for DNA with a cytosine at the second position from the 5′ end (5′-xCx DNA) (54). Moreover, binding of this type of DNA with CpG DNA promotes TLR9 dimerization and activation, suggesting that activation of TLR9 is regulated by binding to two types of DNA. Indeed, co-stimulation of mouse bone marrow-derived macrophages and pDCs with CpG DNA and 5′-xCx DNA increased TLR9 activation (54). It remains to be elucidated whether the recognition of 5′-xCx DNA motif has any advantage in inducing immune responses. Studies using TLR9-deficient mice showed that TLR9 was physiologically involved in sensing DNA viruses, including MCMV, HSV-1, HSV-2, and adenovirus (2).

## Cytosolic RNA Sensor: RLRs

**Figure 2:**
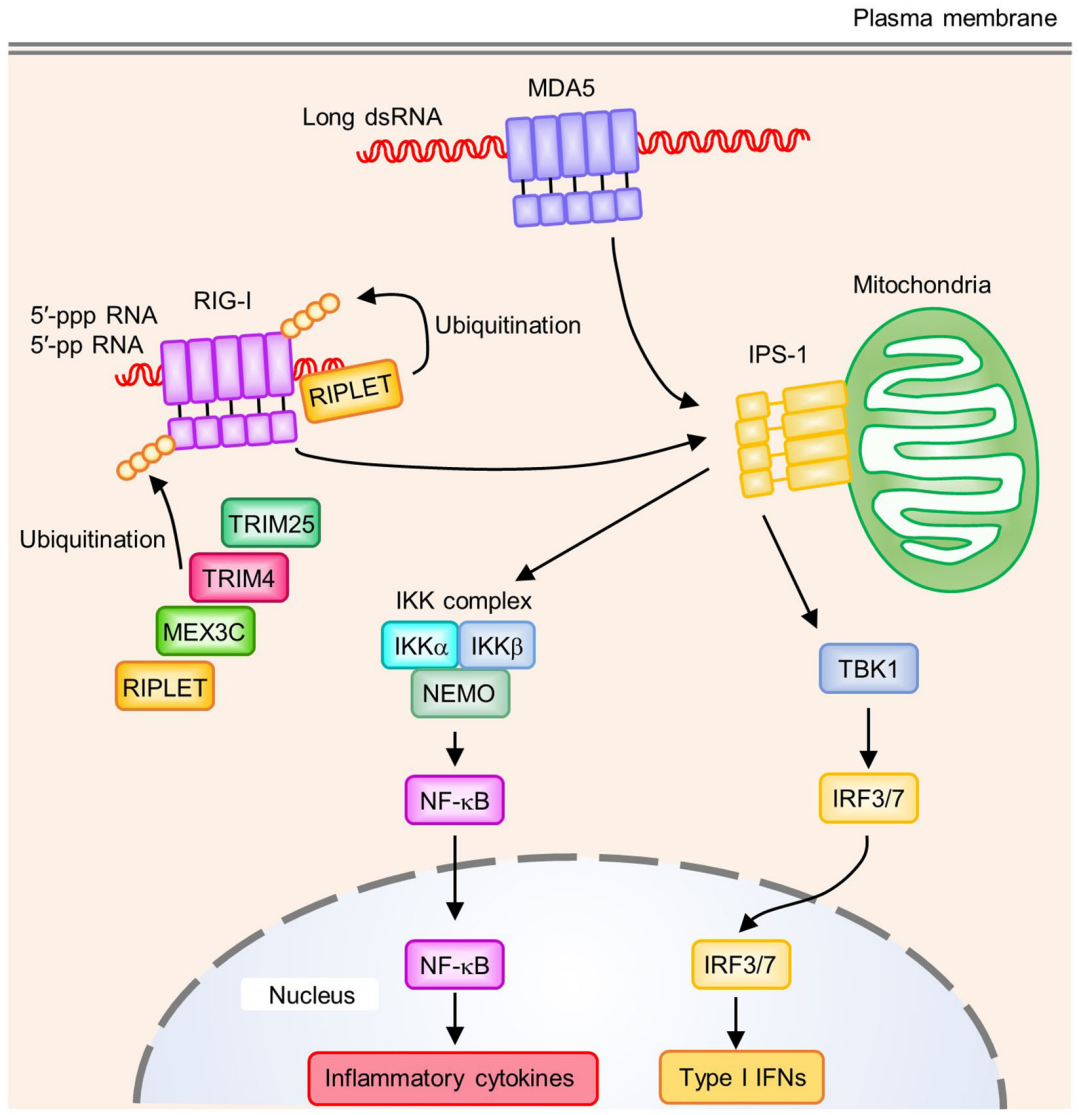
Signaling pathways of RIG-I-like receptors (RLRs). RIG-I recognizes the 5′-tri- or 5′-di-phosphate end of RNA. A blunt-end at the triphosphate end and an unmethylated 5′-terminal nucleotide at the 2′-O position are also required to activate RIG-I. MDA5 binds to long dsRNA, which allows the oligomerization of MDA5 by forming a helical filament-like structure. Polyubiquitination on RIG-I CTD by RIPLET has a critical role in RIG-I activation. In addition to RIPLET, other E3 ubiquitin ligases such as TRIM25, TRIM4, and MEX3C act as positive regulators by mediating K63-linked polyubiquitination on RIG-I CARDs. Oligomerized CARDs of RIG-I or MDA5 interact with IPS-1 on mitochondria, which activates downstream signaling pathways. IPS-1 induces activation of the TBK1 and IKK complex (IKKα, IKKβ, and NEMO), which activates the transcription factors IRF3/7 and NF-κB, respectively. These transcription factors induce the production of type I IFNs and proinflammatory cytokines.

Invading RNA viruses release their RNA into the cytoplasm of host cells and force the host cell to synthesize viral components by using the host machinery. The innate immune system can sense cytosolic RNA *via* RLR family members (**Figure 2**). RLRs are composed of retinoic acid-inducible gene I (RIG-I), melanoma differentiation-associated protein 5 (MDA5), and laboratory of genetics and physiology2 (LGP2), which are upregulated by type I IFN exposure in various tissues (55–58). RLRs share structural features consisting of a central DExD/H box RNA helicase domain and a C-terminal domain (CTD), which sense RNA. In addition, RIG-I and MDA5 have two caspase activation and recruitment domains (CARDs) at the N-terminus that mediate downstream signaling. In contrast, LGP2 lacks CARD, and its physiological function with regard to RIG-I- or MDA5-mediated signaling remains controversial (59, 60).

RIG-I and MDA5 recognize different dsRNA species. RIG-I recognizes relatively short dsRNA while MDA5 preferentially binds to long dsRNA (>1 kb) (61). In addition to RNA length, RIG-I requires additional properties at the dsRNA 5′-end. Although short dsRNA without a 5′-triphosphate was proposed to activate RIG-I, a 5′-tri- or 5′-di-phosphate end in dsRNA seems to be important for the strong activation of RIG-I (61, 62). Furthermore, a blunt-end at the triphosphate end and unmethylated 5′-terminal nucleotide at the 2′-O position were important for RIG-I activation (63, 64). In addition to dsRNA, RIG-I recognizes ssRNA with a 5′-triphosphate to activate downstream signaling pathways (65, 66). However, the length and the degree of complementarity of dsRNA are considered more important for the activation of MDA5 (**Figure 2**) (61, 67). Because host-derived RNA undergoes 5′-processing, including cap formation by 2′-O-methylation in the nucleus, and long dsRNA is not normally present in host cells, these ligand specificities of RIG-I and MDA5 are critical to avoid the recognition of self-derived RNA.

In the steady state, RIG-I is present in an auto-repressed conformation, which masks its CARDs to prevent signal transduction. Binding of nucleic acids to RIG-I leads to an ATP-dependent conformational change, which results in the release of CARDs from autoinhibition (68). This conformational change allows CARDs to undergo additional modifications such as polyubiquitination (**Figure 2**) (55). Covalent conjugation and non-covalent binding of K63-linked polyubiquitin chains to the CARDs of RIG-I lead to the formation and stabilization of a RIG-I-tetramer that functions as a signaling platform (69, 70). Several E3 ubiquitin ligases positively regulate the RIG-I signaling pathway, including TRIM25, RIPLET, TRIM4, and MEX3C (71–74). All of these E3 ligases are involved in the K63-linked polyubiquitination of RIG-I CARDs, while only RIPLET was reported to mediate the K63-linked polyubiquitination of RIG-I CTD (75). This polyubiquitination of RIG-I CTD promotes the release of RIG-I CARD autoinhibition and is required for TRIM25-mediated RIG-I activation, suggesting RIPLET may be a prerequisite for RIG-I activation (75, 76). Recent studies showed that RIPLET, but not TRIM25, is required for RIG-I signaling (77, 78). These findings support the critical role of RIPLET in the RIG-I signaling pathway, and indicate that other E3 ligases for CARD polyubiquitination might be functionally redundant. In addition to ubiquitination, a recent study showed that RIPLET regulated the RIG-I signaling pathway in a ubiquitin-independent manner. RIG-I forms filaments on dsRNA and RIPLET binds to the filamentous oligomers of RIG-I, which induces the cross-bridging of RIG-I filaments and receptor clustering that allows the efficient activation of RIG-I signaling. However, ubiquitination by RIPLET is dispensable for MDA5 activation, which requires the formation of a helical filament along with long dsRNA, allowing the oligomerization of CARDs (79–81). In contrast, ZNF598, another E3 ubiquitin ligase that negatively regulates RIG-I-mediated signaling, interacts with RIG-I to deliver a ubiquitin-like protein FAT10 to RIG-I, which inhibits the K63-linked polyubiquitination of RIG-I and prevents activation of the RIG-I signaling pathway (82).

Oligomerization of the CARDs of RIG-I or MDA5 upon dsRNA recognition induces their interaction with the CARD of adaptor protein interferon-β promotor stimulator 1 (IPS-1, also known as MAVS) (**Figure 2**) (83, 84). In addition to CARD, IPS-1 contains a proline-rich region, TRAF-interacting motifs (TIMs), and a C-terminal transmembrane domain. IPS-1 anchors to the outer mitochondrial membrane (OMM), mitochondrial-associated endoplasmic reticulum membranes (MAMs), and peroxisomes *via* its C-terminal transmembrane domain (85). The binding of IPS-1 to RIG-I or MDA5 leads to the oligomerization of IPS-1 to form prion-like aggregates, which are crucial for activating downstream singling pathways (86). IPS-1 activates TBK1 to induce the IRF3- or IRF7-mediated transcription of type I IFNs, and also the IKK complex (IKKα, IKKβ, NEMO) to induce the NF-κB-mediated transcription of inflammatory cytokines (55, 87).

Although RIG-I and MDA5 sense cytosolic RNA, their responses to RNA viruses are different. RIG-I recognizes Paramyxoviruses, Rhabdoviruses, Orthomyxoviruses, Filoviruses, and Flaviviruses, such as Sendai virus, Newcastle disease virus (NDV), VSV, influenza virus, Ebola, and hepatitis C virus (HCV). In contrast, MDA5 recognizes Picornaviruses, such as EMCV, Theiler’s virus, and Mengo virus. Viruses including dengue virus, WNV, and reovirus are recognized by RIG-I and MDA5 (55). In addition to RNA viruses, several DNA viruses also activate RIG-I and MDA5. Epstein-Barr virus (EBV) and adenovirus stimulate the RIG-I signaling pathway. EBV-encoded small RNAs (EBERs), short noncoding RNAs that are highly abundant viral transcripts in latently EBV-infected cells, are recognized by RIG-I (88). Adenovirus also produces short noncoding RNA called adenovirus-associated RNAs (VA) in infected cells, and VA induce type I IFNs by a RIG-I-dependent mechanism (89). RNA polymerase III (Pol III) is an enzyme that mediates the synthesis of EBERs and VA that contain a 5′-triphosphate from viral DNA. In human primary macrophages, the early induction of type I IFNs against HSV-1 is dependent on MDA5; however, Pol III does not appear to mediate this response (90). MDA5 also induces innate immune signaling against hepatitis B virus (HBV) by associating with HBV-specific nucleic acids (91).

## Cytosolic DNA Sensor: cGAS

**Figure 3:**
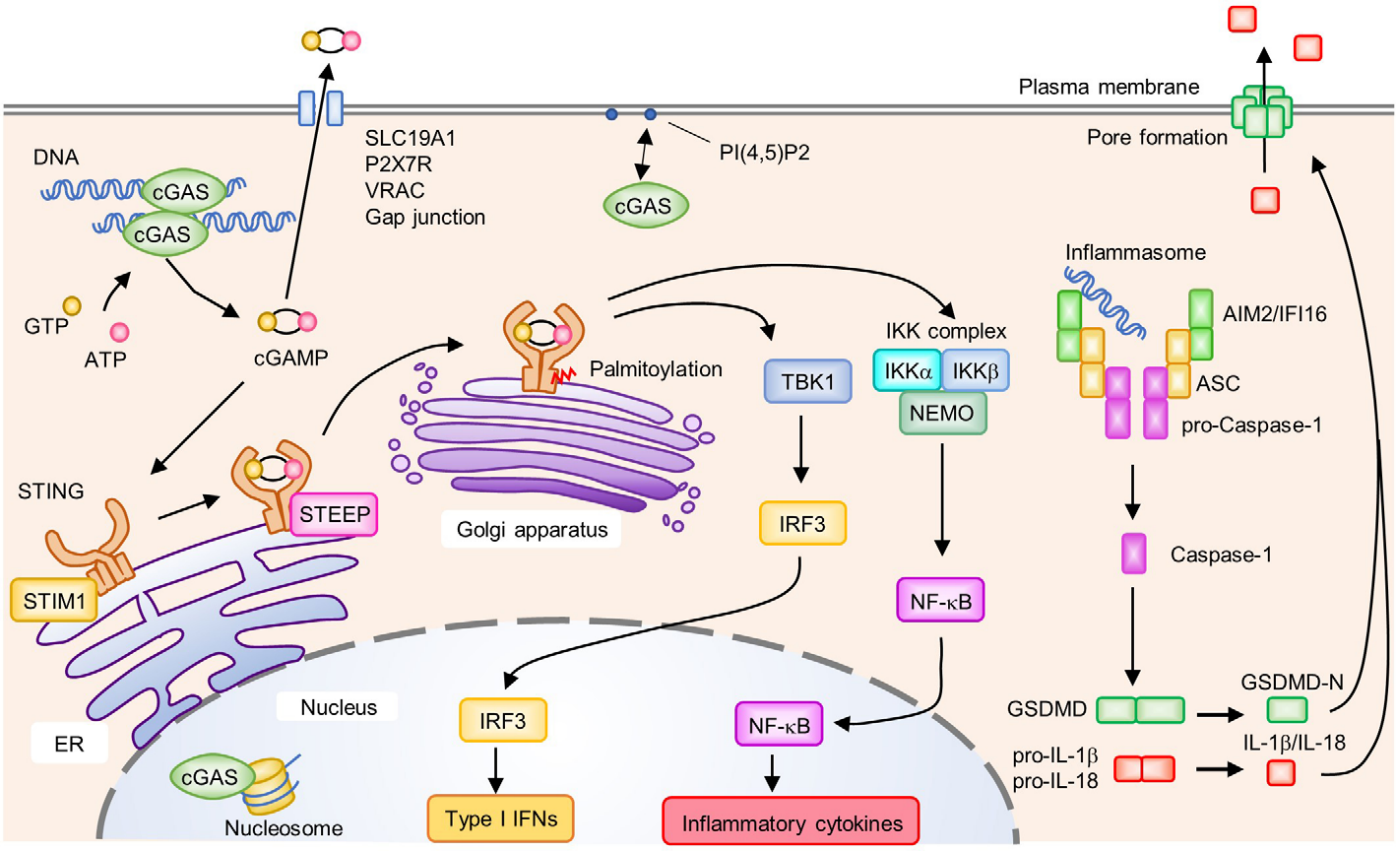
Activation of the cyclic GMP-AMP (cGAMP) synthase (cGAS)-stimulator of IFN genes (STING) signaling pathway and AIM2/IFI16 inflammasome. cGAS recognizes DNA in the cytosol and subsequently synthesizes the second messenger cGAMP from GTP and ATP. In the steady state, STIM1 interacts with STING to retain it on the endoplasmic reticulum (ER) membrane. Binding of cGAMP to STING induces the translocation of STING from the ER to the Golgi apparatus. Upon activation, enhanced interactions between STEEP and STING promote the trafficking of STING from the ER to the Golgi apparatus where it undergoes post-translational modifications such as palmitoylation. Activated STING on the Golgi apparatus recruits and activates TBK1 and the IKK complex (IKKα, IKKβ, and NEMO), which induce the production of type I IFNs and proinflammatory cytokines by IRF3 and NF-κB, respectively. cGAMP activates surrounding cells by being transferred to the extracellular space *via* SLC19A1, P2X7R, VRAC, and gap junctions. cGAS is associated with PI(4,5)P2 on the plasma membrane and is trafficked away from the nucleus to prevent the aberrant activation of cGAS by self-derived DNA. cGAS is also localized in the nucleus where its activity is inhibited by interactions with the nucleosome. Cytosolic DNA is recognized by ALRs, leading to the formation of an inflammasome composed of AIM2 or IFI16, ASC, and pro-Caspase-1. Within the inflammasome, Caspase-1 is activated by proteolytic cleavage from pro-Caspase-1 to Caspase-1. Activated Caspase-1 cleaves GSDMD, pro-IL-1β; and pro-IL-18. The N-terminus of GSDMD (GSDMD-N) forms a pore on the plasma membrane and induces cell death accompanied by the release of biologically active IL-1β and IL-18.

Z-DNA binding protein 1 (ZBP1), IFN-gamma inducible protein 16 (IFI16), Pol III, MRE11, and cyclic GMP-AMP (cGAMP) synthase (cGAS) were reported to be cytosolic DNA sensors that induce type I IFNs (55, 92–96). Absent in melanoma 2 (AIM2) is a cytosolic DNA sensor that induces caspase-1-dependent IL-1β production and pyroptotic cell death rather than type I IFNs (discussed below). Among these molecules, cGAS, an enzyme that synthesizes the second messenger cGAMP from ATP and GTP upon its binding to dsDNA, plays a central role in recognizing cytosolic DNA, which induces the production of type I IFNs and proinflammatory cytokines (**Figure 3**). cGAS binds to dsDNA independent of its sequence by forming a 2:2 cGAS-dsDNA complex (97, 98). However, the length or bending of dsDNA seems to be a key factor for cGAS activation. Furthermore, compared to short dsDNA, long dsDNA is a potent activator of cGAS (99, 100). Long and bent dsDNA allows cGAS dimers to form protein-DNA ladder-like structures, which stabilize complexes consisting of two cGASs and two dsDNAs (99). Mitochondrial transcription factor A (TFAM) and high-mobility group box 1 protein (HMGB1) are known as endogenous DNA-interacting proteins that are able to induce U-turns and bends in DNA, which nucleate the formation of cGAS dimers to enhance the activation of cGAS. In addition to its dimerization, the length of dsDNA influences the efficiency of signal transduction (101).

cGAS was reported to localize to plasma membrane, cytosol, and nucleus (95, 102–104). Depending on cell types, the localization of cGAS is different (102). Furthermore, the localization of cGAS might change upon DNA stimulation (102). In mouse and human phagocytes, cGAS interacts with PI(4,5)P2, a phosphoinositide in the plasma membrane, to promote its localization to the plasma membrane, which may prevent excessive immune responses to self-derived dsDNA, which is abundant in the nucleus (**Figure 3**) (102). However, recent reports suggested that cGAS is localized in part in the nucleus (103, 104). Although cGAS is expressed as a cytosolic protein, it can bind to self-derived dsDNA when the nuclear envelope undergoes breakdown during the cell cycle and generates daughter cells that contain cGAS in the nucleus. However, the activity of cGAS in response to self-derived dsDNA is less than for exogenous dsDNA although nuclear-localized cGAS can also induce innate immune signaling. Therefore, in addition to the existence of nuclear envelope, there may be an unknown regulatory mechanism which prevents the activation of immune response against self-DNA. Recently, structural analyses of the complex formed between nucleosome core particles (NCPs) and cGAS revealed that the nucleosome inhibits cGAS activation by binding to the DNA binding site of cGAS to prevent its dimerization by steric hindrance with the proximal NCPs (105–109). In the presence of DNAs and nucleosomes, cGAS preferentially binds to nucleosomes, which might be a key regulatory strategy allowing cGAS to localize to the nucleus without persistent activation. Positively charged residues of human and mouse cGAS, such as lysine and arginine, were reported to be important for their specific binding to the acidic part of nucleosomes, and mutations in these positions disrupt the interaction with nucleosomes to abolish the cGAS-suppressive effect of NCPs. Moreover, nuclear cGAS accelerates irradiation-induced genome destabilization and cell death by restraining homologous recombinant-DNA repair. This inhibition is achieved by the compression of dsDNA to a higher order state through its binding to dsDNA and self-oligomerization (103). Thus, cGAS regulates various cellular responses in which nuclear self-derived DNA is involved, in addition to antiviral innate immune responses to foreign DNA.

Upon activation, cGAS produces the second messenger cGAMP from ATP and GTP, which subsequently binds to and activates ER-resident adaptor protein stimulator of IFN genes (STING), resulting in a conformational change and oligomerization of STING (**Figure 3**) (110, 111). Of note, cGAMP can be transferred to surrounding cells *via* SLC19A1, P2X7R, or LRRC8A/LRRC8E-containing volume-regulated anion channels and gap junctions, inducing the STING-dependent production of type I IFNs in neighboring cells (112–116). In addition, cGAMP can be incorporated into viral particles and newly formed viruses transmit antiviral signals to subsequently infected cells (117, 118). Such cell-to-cell transfer of cGAMP promotes the rapid propagation of inflammatory signals and the amplification of inflammatory responses.

Upon DNA stimulation, STING changes its cellular localization from the ER to the Golgi apparatus *via* ER-Golgi intermediate compartments, which is necessary to activate the downstream signaling pathway (**Figure 3**) (119). In the inactivated state, STING is retained on the ER membrane by its association with ER-resident protein stromal interaction molecule 1 (STIM1). The binding of cGAMP to STING reduces the association between STIM1 and STING, and promotes its translocation to the Golgi apparatus (120). TOLLIP, another protein that interacts with resting-state STING, stabilizes STING by preventing its degradation by the lysosome pathway (121). Knockout of TOLLIP ameliorates autoimmune symptoms in Trex1-knockout mice in which cGAS-STING-mediated signaling is activated (121). A recent study showed that CxORF56, also known as STING ER exit protein (STEEP), interacted with STING to promote its trafficking from the ER to the Golgi apparatus. STEEP augments PI(3)P accumulation in the ER and promotes ER membrane curvature, which facilitates COPII-mediated STING ER exit (122). In contrast, myotubularin-related protein 3 (MTMR3) and MTMR4, members of the phosphatase superfamily, negatively regulate STING-mediated innate immune responses by reducing PI(3)P levels. MTMR3 and MTMR4 deficiencies resulted in increased PI(3)P and rapid STING trafficking from the ER to the Golgi apparatus upon DNA stimulation (123). Post-translational modifications such as phosphorylation and ubiquitination are also involved in STING activation (124, 125). In addition, the palmitoylation of STING by palmitoyl transferases (DHHC3, DHHC7, and DHHC15) in the Golgi apparatus is necessary for STING-dependent IFN production (126, 127). Cysteine residues in proteins are the target sites for palmitoylation, and Cys88/91 on STING is thought to be critical for its modification and activation. Activated STING subsequently recruits and activates TBK1 to phosphorylate STING at a pLxIS motif (31, 128). This further induces IRF3 recruitment, and in turn, TBK1 phosphorylates IRF3, leading to type I IFN expression. A small GTPase RAB2B and its effector protein Golgi-associated RAB2B interactor-like 5 (GARIL5) were reported to positively regulate the cGAS-STING signaling pathway (129). The RAB2B-GARIL5 complex colocalizes with STING on the Golgi apparatus to regulate the cGAS-STING signaling pathway by promoting the phosphorylation of IRF3 by TBK1 (129). STING also activates the IKK complex to induce the translocation of NF-κB into the nucleus (**Figure 3**). Notably, cGAS-STING pathway also induces autophagy, which is thought to play a role in mediating the clearance of cytosolic DNA or DNA viruses (130).

A number of studies using cGAS- or STING-deficient mice showed that cGAS is involved in antiviral responses against a wide range of DNA viruses, including HSV, vaccinia virus, and murine gamma herpesvirus 68 (MHV68) (131). Importantly, retroviruses including HIV also activate the cGAS-STING pathway. Following retroviral infection, cGAS recognizes DNA, reverse-transcribed from viral genomic RNA, which is incorporated into the host cell genome (132). In addition to viruses, cGAS is also involved in immune responses against intracellular bacteria, such as *Listeria monocytogenes* and *Neisseria gonorrhoeae (* 133, 134). Interestingly, certain bacteria can activate cGAS indirectly by inducing cellular stress. For example, *Mycobacterium tuberculosis* causes the release of mitochondrial DNA (mtDNA) into the cytoplasm from mitochondria, which activates cGAS (135). A recent study reported that cell fusion induced by bacteria, such as *Burkholderia pseudomallei*, acts as a danger signal in the host and triggers genomic instability and micronuclei formation, resulting in cGAS activation. This activation of cGAS leads to autophagic cell death rather than type I IFN production (136). cGAS can also recognize mtDNA released through Bak- and Bax-mediated pore formation during apoptosis (137). However, the activation of cGAS-STING pathway in apoptotic cells is inhibited by pro-apoptotic caspases which cleave key proteins for production of type I IFNs, including cGAS and IRF3, to prevent inflammation induced by cell death (137–139).

## Cell Death Induced by Nucleic Acid Sensors

Nucleic acid sensors are shown to trigger cell death such as apoptosis, pyroptosis and necroptosis. RLRs induce apoptosis *via* IRF3 (140). Activated IRF3 interacts with Bax, a pro-apoptotic protein, which induces their co-translocation to mitochondria and triggers Cytochrome c release to cytoplasm (140). This IRF3-mediated apoptosis pathway is not dependent of transcriptional activation of IRF3, but linear polyubiquitination of IRF3 by the protein complex, LUBAC (linear ubiquitin chain assembly complex) (141). IRF3-mediated apoptosis is sufficient for protection against pathogenesis in Sendai virus infection (141). Activation of TLR3-TRIF pathway also induces apoptosis in cancer cells. TRIF interacts with RIPK1 through their RIP homotypic interaction motif (RHIM) domains and forms a complex with caspase-8 to induce apoptosis in the absence of cellular inhibitor of apoptosis proteins (cIAPs) (142).

AIM2-like receptors (ALRs), including AIM2 and IFI16, recognize cytosolic DNA and induce inflammatory responses. The recognition of DNA by ALRs promotes the formation of an inflammasome, a multiprotein complex formed in response to pathogens and endogenous danger signals, leading to a programmed, immunogenic, and lytic type of cell death termed pyroptosis (143). The inflammasome activates Caspase-1 (proteolytic cleavage from pro-Caspase-1 to Caspase-1), resulting in maturation of the inflammatory cytokines IL-1β and IL-18, as well as the cleavage of Gasdermin D (GSDMD). Subsequently, the N-terminus of cleaved GSDMD form a pore at the plasma membrane that leads to pyroptosis accompanied by the release of biologically active cytokines (IL-1β and IL-18) (**Figure 3**). However, in human monocytes, the inflammasome activation by cytosolic DNA is dependent on cGAS-STING-NLRP3 axis, but not AIM2 (144). Mechanistically, STING triggers lysosome membrane permeation, which results in NLRP3 inflammasome activation. Moreover, cGAMP contributes to NLRP3 and AIM2 inflammasomes activation in bone marrow-derived macrophages (145). cGAS-STING-NLRP3 axis is activated upon HSV-1 infection (146). HSV-1 infection promotes the STING-NLRP3 interaction and facilitated the formation NLRP3 inflammasome in ER.

The AIM2 inflammasome is involved in responses against viral and bacterial infections. Several bacterial species were reported to activate AIM2 including *Francisella tularensis*, *Listeria monocytogenes*, *Streptococcus pneumoniae*, *Brucella abortus*, and *Chlamydia muridarum (* 147, 148). Because AIM2 is localized in the cytoplasm, bacterial DNA must be released into the cytoplasm for AIM2 to access it. This is achieved by guanylate-binding proteins (GBPs) that are involved in bacteriolysis and which are important for AIM2 inflammasome activation. Indeed, *Francisella novicida* infection induces the expression of GBP2 and GBP5 in the cytosol, which is dependent on the IRF1-mediated induction of type I IFNs. These proteins associate with *Francisella novicida* in the cytoplasm to trigger bacteriolysis, which allows AIM2 to recognize dsDNA released from bacteria (149, 150). Furthermore, another IFN inducible gene, interferon response gene B10 (IRGB10), is associated with these GBPs, and in combination they induce the membrane rupture of *Francisella novicida (* 151). Moreover, AIM2 inflammasome is activated by several DNA viruses, such as MCVM, vaccinia virus, and human papillomavirus (147, 148, 152). In addition to viral DNA, AIM2 recognizes self-derived DNA released from tissues damaged by viral infection. IAV was reported to trigger the release of mitochondrial or nuclear DNA from macrophages and damaged lung tissues, leading to AIM2 inflammasome activation. However, it is debatable whether this activation of AIM2 is protective or harmful (153–155).

IFI16 is localized in the cytosol and nucleus, and is associated with the production of type I IFNs and cell death induced by HSV, HIV-1, Kaposi sarcoma-associated herpesvirus (KSHV), and intracellular bacterium *Listeria monocytogenes (* 94, 133, 156–158). Furthermore, in human cells, IFI16 is thought to cooperate with the cGAS-STING pathway to induce robust host anti-viral responses (156, 157). In contrast, STING negatively regulates IFI16 by recruiting an E3 ligase TRIM21 to induce its degradation (159). This STING-mediated negative feedback might prevent excess immune responses mediated by IFI16. IFI16 is localized in the nucleus and acts as a nuclear sensor for nuclear replicating viruses such as KSHV, EBV, and HSV-1 (158). A previous study reported that IFI16 colocalized with virus genomes in the nucleus to form an inflammasome complex, which is then relocated into the cytoplasm to induce inflammasome activation and STING-dependent IFN responses. Furthermore, breast cancer 1 (BRCA1), a DNA damage repair sensor and transcription regulator, was reported to be a positive regulator of IFI16. BRCA1 interacts with IFI16 in the nucleus, and is enhanced upon virus infection. The knockdown of BRCA1 decreased the association of IFI16 with the viral genome and reduced the subsequent activation of inflammasomes and IFN responses (160).

TLR3 and ZBP1 induce necroptosis, a lytic type of cell death which is regulated by receptor-interacting protein kinase 3 (RIPK3) and mixed-lineage kinase domain-like pseudokinase (MLKL). RIPK3 is activated by RIPK1 which is activated by death receptors, such as TNF receptor 1 (TNFR1), CD95, and TRAIL-R, when caspase-8 activation is inhibited (161). Activated RIPK3 phosphorylates MLKL, which in turn triggers MLKL oligomerization, membrane translocation, and membrane disruption. In addition to RIPK1, the activation of RIPK3 is mediated by other RHIM domain-containing molecules. TRIF and ZBP1, which contain the RHIM domain, also induce necroptosis by interacting with RIPK3 (162, 163).

## Roles of Nucleic Acid Sensors in Inflammatory Diseases

### Autoinflammatory Diseases Caused by Aberrant Activation of Nucleic Acid-Sensing Pathways

Although the induction of inflammatory responses through the above mentioned nucleic acid receptors is important for protecting hosts from invading pathogens, autoinflammatory pathology can be caused by aberrant inflammatory responses, specifically abnormalities in receptors, signaling molecules, and nucleic acid metabolism. Mutations in the RNA helicase domains of RIG-I and MDA5 were found in patients with systemic lupus erythematosus (SLE), Aicardi-Goutieres syndrome (AGS), and Singleton-Merten syndrome (SMS), all of which exhibit a type I IFN signature (164–169). A mutation in RIG-I was reported in SMS whereas mutations in MDA5 were associated with the various diseases described above. Mice with an *Ifih1* missense mutation encoding MDA5, developed lupus-like autoimmune symptoms without viral infection (170). TLR7 and TLR9 were reported to be involved in the pathogenesis of SLE. The proportions of B cells and monocytes expressing TLR9 were higher among patients with active SLE than among patients with inactive SLE, and this correlated with the presence of anti-dsDNA antibodies (171). Another study showed that mice overexpressing TLR7 developed SLE-like disease (172). In addition, IFNα production mediated by TLR7 was increased in pDCs derived from SLE patients, and correlated with disease severity. Furthermore, enhanced IFNα production was associated with increased TLR7 expression in the late endosomal/lysosomal compartment in lupus pDCs (173).

Genetic mutations in molecules that function in signaling cascades downstream of nucleic acid sensors also cause autoinflammatory diseases. STING gain-of-function mutations (V147L, N154S, and V155M) are involved in lupus-like syndromes and STING-associated vasculopathy with onset in infancy (SAVI) (174, 175). SAVI is characterized by systemic inflammation, interstitial lung disease, cutaneous vasculitis, and recurrent bacterial infection. The STING mutation, STING-V155M, which is localized mainly in the Golgi apparatus and perinuclear vesicles in fibroblasts independent of the presence of its ligand, interacts with STEEP to a greater degree compared with WT STING (122, 176). Recently, it was reported that C9orf72 is essential for control of immune activation mediated by STING and the loss of C9orf72 promoted the production of type I IFNs (177). Expansion of a hexanucleotide repeat (GGGGCC) in the *C9orf72* gene was shown to be the major cause of familial amyotrophic lateral sclerosis (ALS) and frontotemporal dementia (FTD). Blood monocyte-derived macrophages from these patients showed an enhanced type I IFN signature (177). Furthermore, IRF5, a downstream mediator of TLR signaling, was also identified as an autoimmune susceptibility gene (178). IRF5 expression is upregulated in SLE patients and this enhanced expression was associated with the risk haplotype of IRF5 (179). IRF5-deficient mice or SLE model mice treated with an IRF5 inhibitor attenuated lupus pathology (180, 181). *Ex vivo* human studies demonstrated that an IRF5 inhibitor blocked SLE serum-induced IRF5 activation in healthy immune cells and significantly reduced basal IRF5 hyper-activation in SLE immune cells (181).

### Inflammatory Diseases Caused by Dysregulated Nucleic Acid Homeostasis

Molecules involved in nucleic acid metabolism, such as DNases and RNases, play important roles in avoiding aberrant induction of inflammatory responses against self-derived nucleic acids that lead to autoinflammatory diseases.

TREX1, RNase H2 complex, SAMHD1, DNASE1L3, and DNase II are key enzymes that control the turnover of endogenous DNA, and mutations in these genes cause autoinflammatory diseases (182–184). TREX1 is the major mammalian 3′ to 5′ DNA exonuclease located on the ER membrane. Loss-of-function mutations in the human *TREX1* gene were reported to induce AGS and SLE (185, 186). Mutations in *TREX1* cause the accumulation of cellular nucleic acids, and failure to remove these nucleic acids may result in the excessive activation of immune responses against them. Indeed, single-stranded DNA fragments derived from endogenous retroelements that had accumulated in the heart cells of TREX1-deficient mice might induce type I IFN responses (187). An RNase H2 complex comprised of three proteins encoded by *RNASEH2A*, *RNASEH2B*, and *RNASEH2C*, degrades the RNA strand of the RNA-DNA heteroduplex. Mutations in RNase H2 subunits result in genome instability, which causes AGS (188, 189). SAMHD1, a deoxynucleotide triphosphate (dNTP) triphosphohydrolase, is required to maintain the balance of the dNTP pool in cells. Recently, it was reported that SAMHD1 promoted the degradation of nascent DNA at stalled replication forks by activating MRE11 exonuclease independent of the dNTPase activity of SAMHD1, and that SAMHD1-deficiency caused the accumulation of ssDNA in the cytoplasm (190). Mutations in SAMHD1 caused AGS possibly by the accumulation of ssDNA and a genome instability due to the increase in dNTP pools (190, 191). Importantly, enhanced type I IFN production in AGS caused by TREX1, RNaseH2 complex, or SAMHD1 mutations is mediated by the cGAS-STING pathway (190, 192, 193). Of note, genome instability leads to the formation of micronuclei, small DNA-containing structures that are not incorporated correctly into the nucleus after cell division (194). Micronuclear envelopes are prone to rupture, resulting in the release of damaged DNA to the cytosol, which in turn stimulates the cGAS-STING axis to induce inflammatory responses (195, 196). Genome instability triggers the generation of micronuclei, which are thought to promote excessive cGAS-dependent immune responses in cells carrying these mutations (197). DNASE1L3 is a secreted DNase that digests cell-free DNA and chromatin in microparticles derived from apoptotic cells (198, 199). Loss-of-function mutation in *DNASE1L3* leads to rare form of SLE (183). In Dnase1l3-deficient mice, TLR9 and TLR7, but not cGAS-STING pathway, were redundantly required for autoimmunity (199, 200). DNase II is localized in lysosome and digests the DNA from apoptotic cells and nuclear DNA expelled from erythroid precursor cells (201, 202). The embryonic lethality of DNase II knockout mice is rescued by lack of *Ifnar1* gene, suggesting that abnormal activation of type I IFN responses is taken place in this mice (203). Mice lacking DNase II and Ifnar1 developed chronic polyarthritis, and loss of DNase II gene in the bone marrow-derived cell was sufficient to induce this arthritis (184, 204).

To prevent excessive inflammation against self-derived RNA, proper RNA-processing systems is also important. Up to 25% of cytosolic Alu RNAs are forming Alu-Alu hybrids which have duplex RNA structures formed by inverted repeat Alu elements (67). These Alu-Alu hybrids are modified by ADAR1, an adenosine-to-inosine editing enzyme of dsRNA, which results in destabilization of RNA duplexes and prevents the recognition by MDA5 (67, 205, 206). Mutations in ADAR1 cause AGS with aberrant type I IFN response (207). mtRNAs also form double-stranded RNA structures, which can activate MDA5 when they escape to the cytoplasm (208). To restrict the levels of mitochondrial dsRNA, mitochondrial RNA helicase SUV3 and polynucleotide phosphorylase PNPase play an important role (208). Knockdown of PNPase, but not SUV3, caused the release of mitochondrial dsRNA into cytoplasm, and increased type I IFN production through the MDA5-IPS1 axis (208). Mutations in PNPT1, which encodes PNPase, cause several disorders including hearing loss and Leigh syndrome (209, 210).

### Inflammation Induced by Self-Derived Nucleic Acid Recognition in Other Common Diseases

In addition to autoinflammatory diseases, the pathologies of several common diseases are linked to inflammatory responses induced by self-derived DNA or RNA. The cGAS-STING signaling pathway is activated following myocardial infarction by recognizing self-DNA derived from dead cells in the heart. The genetic or pharmacological disruption of cGAS-STING and type I IFN signaling improved survival and pathological remodeling in a myocardial infarction mouse model (211, 212). Parkinson’s disease is also linked to inflammation induced by self-derived DNA. Mutations in Parkin or PINK1, which are involved in mitophagy that removes damaged mitochondria, lead to Parkinson’s disease in humans. The accumulation of damaged mitochondria and increased circulating mtDNA in serum were observed in *Prkn^−/−^* or *Pink1*-knockout mice under mitochondrial stress, and this induced strong inflammation which was rescued by a loss of STING (213). The upregulation of cGAS was observed in the striatum from postmortem Huntington disease (HD) patients, and HD cells showed enhanced inflammatory gene expression and autophagy induction. Numerous micronuclei were found in HD cells indicating they might enhance cGAS activity, which may contribute to HD pathology (214). Psoriasis is another disease in which DNA-induced inflammation is involved. Increased cell-free DNA and mtDNA were detected in the serum of psoriatic patients (215, 216). The topical skin application of imiquimod, a TLR7 ligand, is used to induce psoriasis in mice. In this imiquimod-induced psoriasis model, TLR7 and TLR9, but not TLR7 or TLR9 alone, are required for its pathogenesis, suggesting DNA recognition is important for the development of disease (217). In addition to the activation of TLR7 signaling pathway, imiquimod induces cell death, and thus DNA derived from dead cells might be a trigger of TLR9 signaling in this model (218). LL37, an antimicrobial peptide, has an important role in psoriasis by forming a complex with DNA and delivering cell-free DNA into endosomes, which activates TLR9 (219). Recently, it was reported that topical treatment with cationic nanoparticles, which interfere the DNA-LL37 complex, relieved the symptoms of psoriasis in mice and monkeys (220). Self-RNA-mediated inflammation is also thought to be involved in the pathogenesis of psoriasis. LL37 was reported to form complexes with self-derived RNA as well as self-derived DNA, triggering TLR7 and TLR8 activation in human DCs, which may be associated with psoriasis pathogenesis (221). Another study showed that together with cargo peptides, polyamines form a complex with RNA, promoting endosomal uptake and activation of TLR7 in DCs (222). The decreased expression of protein phosphatase 6 was observed in psoriatic lesions, leading to an increased generation of arginase-1-mediated polyamine. Thus, inflammatory responses induced by self-derived nucleic acids may cause disease as well as contribute to the exacerbation of disease pathogenesis, and the inhibition of nucleic acid-induced inflammation might be a therapeutic target for the treatment of various diseases.

## Nucleic Acid Sensors in Cancer

### Role of Nucleic Acid Sensors in Anti-Cancer Treatment

Many studies have demonstrated the involvement of DNA-sensing pathways in antitumor responses as well as tumor development. Cancer cells often contain cytosolic DNA, which may not be present under physiological conditions. The generation of micronuclei and the release of mtDNA from mitochondria, caused by chromosomal instability and mitochondrial damage, respectively, are the main sources of cytoplasmic DNA in cancer cells. Sensing tumor DNA in tumor cells results in type I IFN production, which contributes to the maturation of DCs and the activation of CD8^+^ T cells that have potent antitumor activity (223). Several reports have suggested the importance of the cGAS-STING axis in antitumor responses, rather than other nucleic acid-sensing receptor-mediated pathways, by bridging innate immune responses and tumor-specific T cell responses *via* the production of type I IFNs. Downregulation of the cGAS-STING pathway in tumors correlates with a poor prognosis in patients with gastric cancer (224). Of note, several human colon cancer cell lines show low or defective STING-mediated signaling, and STING-deficiency in prostate cancer cells increased tumor growth *in vivo (* 225, 226). These defects in the STING pathway may be related to epigenetic silencing *via* methylation of the promoter region of cGAS and STING, or the loss-of-function mutations of these genes (227).

In addition to cancer cells, activation of cGAS-STING pathway in immune cells also contributes to antitumor activities. It is well known that antitumor effects are associated with the production of type I IFNs by DNA sensing after radiotherapy and chemotherapy, which induce DNA damage in cancer cells and the release of DNA into the cytosol (228). The cGAS-STING pathway can be activated by tumor cell-derived DNA, and STING- or IRF3-deficient mice showed defects in priming CD8^+^ T cells and tumor control (113, 229). Furthermore, cancer cell-derived cGAMP is thought to be transferred to neighboring immune cells, resulting in activation of the STING pathway (113, 230). Moreover, tumor-derived DNA is also thought to be transferred to host immune cells and activate immune responses. Treatment with the anticancer drug topotecan induces the release of exosomes containing DNA, which are then taken up by DCs and presented to activate antitumor immunity *via* the STING pathway (231).

Because of the importance of the cGAS-STING pathway in antitumor activities, cyclic dinucleotide (CDN), a STING agonist structurally related to cGAMP, is thought to be useful for anti-cancer therapy. Indeed, treatment with the STING agonist cGAMP inhibited tumor growth in mice (232, 233). STING-activating nanoparticles containing cGAMP were designed to enhance the efficacy of CDN by protecting it from clearance and increasing its transport to the cytosol, and nanoparticle treatment of mice injected with poorly immunogenic B16.F10 melanoma showed a decreased tumor growth rate and prolonged survival relative to mice treated with pure cGAMP (234). The antitumor efficacy of cGAMP treatment was further enhanced with anti-CTLA4 and PD-1 immune checkpoint blockade (233, 234). Based on their anticancer activities in mice, synthetic CDNs that stimulate STING have been approved for clinical trials to test their anticancer effects in humans (235).

In addition to DNA sensors, RNA sensors also contribute to the elimination of cancer cells. DNA methyltransferase inhibitors have been shown to exert clinical anti-tumor effect by inducing MDA5 and TLR3 signaling pathways (236, 237). The activation of these RNA sensors might be induced by dsRNAs derived from endogenous retroviruses (ERVs) which are normally silenced in cells by DNA methylation (237). Furthermore, ablation of histone H3K4 demethylase LSD1 resulted in upregulation of ERVs and accumulation of dsRNAs that are recognized by MDA5 and TLR3 in cancer cells, which promotes anti-tumor T cell immunity and elicits significant responses to anti-PD-1 therapy in a mouse melanoma model (238). Therefore, inhibition of LSD1 might also be a potent strategy in cancer immunotherapy. Radiation therapy also induces the activation of ERVs, which mediates IPS-1-dependent type I IFN response in A549 and B16F10 cells (239). Another study suggests that RIG-I but not MDA5 is important in inducing IFN signaling and cytotoxic effects in response to radiation therapy in cancer cells, such as human D54 glioblastoma and HCT116 colorectal carcinoma cells (240). In these cells, accumulation of U1 non-coding RNA (ncRNA) in the cytoplasm was observed following radiation, suggesting that this ncRNA may contribute to activate RIG-I signaling pathway (240). These differences in radiation-induced responses may vary according to the strength of radiation or types of cancer cells. Endogenous dsRNAs which are originated from pre-mRNA introns were also reported to induce anti-tumor effects. Heterogeneous nuclear ribonucleoprotein C (HNRNPC) is up-regulated in multiple tumors or tumor cell lines. Knock-down of HNRNPC in breast cancer cells, such as MCF7 and T47D, results in accumulation of endogenous dsRNAs which are largely from Alu introns, and induces tumor-inhibitory effect by activating RIG-I-mediated type I IFN responses (241). Argonaute 1x (AGO1x), a translational read-through isoform of AGO1, is also highly expressed in breast cancer cells and is involved in the responses to endogenous dsRNAs (242). AGO1x interacts with dsRNA-processing proteins such as PNPT1 and this complex prevents the accumulation of dsRNAs in cells. Genetic deletion of AGO1x results in dsRNA accumulation and increased IFN responses (242). Deletion of ADAR1, an adenosine deaminase that limits the sensing of endogenous dsRNA, also induces MDA5-dependent type I IFN production and inflammation, which increase the sensitivity of tumors to radiation therapy and immunotherapy (243). The activation of innate immune cells by ligands of endosomal TLRs is another strategy for antitumor therapy (244, 245). TLR ligands are often studied for their effectiveness as adjuvants to induce antitumor T cell activity. The application of liposomes loaded with tumor-specific synthetic peptides and poly(I:C) induced tumor regression and controlled the outgrowth of melanoma and human papillomavirus-induced tumors (245). Another study showed that the administration of ARNAX, a TLR3-specific adjuvant, with a tumor-specific antigen promoted tumor regression. When in combination with anti-PD-L1, this cocktail led to the relief of anti-PD-1 unresponsiveness (244).

### Role of Nucleic Acid Sensors in Tumor Growth

Although there are many reports of cGAS-STING pathway for anti-tumor effects, it was also reported that activation of the cGAS-STING axis in metastatic cancer caused chronic inflammation in tumor tissues, which enhanced cancer cell survival and metastasis. The transfer of cGAMP from cancer cells to astrocytes through gap junctions promoted the production of type I IFNs and proinflammatory cytokines, which in turn supported the brain metastasis of cancer cells (246). While canonical NF-κB signaling is required for antitumor immunity, the noncanonical NF-κB signaling pathway was reported to negatively regulate radiation-induced antitumor immunity (247). In metastatic cells, chromosomal instability is enriched compared with primary tumors, and this leads to activation of the STING-dependent noncanonical NF-κB signaling rather than canonical NF-κB and IRF3 signaling (248). Given that the cGAS-STING pathway can be beneficial and harmful in terms of antitumor immunity, the future direction of therapeutic strategies involving the cGAS-STING pathway should consider the efficiency and safety concerns of the treatment in different stages and type of cancers.

In addition to the cGAS-STING pathway, it was reported that activation of RIG-I signaling pathway in breast cancer cells also enhanced tumor growth, metastasis, and therapy resistance. Cancer cells interact and activate stromal cells to enhance RN7SL1 RNA levels by pol III, which results in secretion of exosomes containing RN7SL1 (249). This exosome activates RIG-I in breast cancer cells and leads to cancer progression.

Endosomal TLRs may also play a role in tumor progression. Mutations in MyD88 (L265P) are frequently (90% of cases) found in Waldenström’s macroglobulinemia (WM), a lymphoplasmacytic lymphoma characterized by an excess of IgM-secreting lymphoplasmacytic cells in the bone marrow (250). MyD88-L265P spontaneously activates the Myddosome, resulting in the constitutive production of proinflammatory cytokines. Combined TLR3/7/9 deficiency was reported to induce tumor regression dependent on the activities of CD4^+^ and CD8^+^ T cells (251). Moreover, activation of endothelial TLR3 by the detection of extracellular RNA from tumors enhanced metastatic progression. TLR3 activation induced the expression of the axon guidance gene SLIT2 in endothelial cells, which mediated the migration of cancer cells to endothelial cells for intravasation, which was dependent on ROBO1, a SLIT2 receptor (252). The detection of tumor-derived exosomal RNAs by TLR3 in lung epithelial cells might also be involved in tumorigenesis. Activation of lung epithelial TLR3 induced neutrophil recruitment to the lungs and lung metastatic niche formation (253).

## Conclusions

Numerous studies have reported detailed mechanisms for ligand recognition and activation by PRRs. Because all pathogens possess nucleic acids, a defensive barrier network consisting of PRRs recognizing pathogenic DNA or RNA as PAMPs and their downstream signaling pathways is important for host protection against invading pathogens such as viruses and bacteria. However, host cells also possess nucleic acids. Therefore, the recognition system for nucleic acids must be strictly controlled regardless of whether nucleic acids are derived from either the host or the invading pathogen. Indeed, the activation of PRRs and downstream signaling pathways are strictly regulated at multiple steps including cellular localization, post-translational modifications, and binding by inhibitors and activators. Nucleic acid metabolism is also critical for limiting responses to self-derived nucleic acids, and defects in the regulatory mechanism lead to autoinflammatory diseases. Therefore, it is important to investigate how nucleic acid-mediated signaling is activated and terminated. A number of negative regulators have been reported to date, and it will be a topic for future research that should be uncover the detailed mechanisms of how and under what conditions such negative regulators function.

Host immune responses induced by nucleic acids are a double-edged sword for the host. Even if immune responses are physiologically induced in response to invading pathogens or damaged cells as host defense, they can lead to morbid symptoms as side effects, and at worst, death. Therefore, the inhibition of nucleic acid-sensing receptors and their signaling pathways might be a promising treatment for undesired and severe inflammatory conditions. Nevertheless, the activation of nucleic acid-induced signaling pathways, especially the STING pathway, may enhance therapeutic effects in cancer, although the effect of the treatment is dependent on the stage and type of cancer. Coupled with the advances in immune checkpoint blockade therapy, it is expected that CDN-based therapy will be used in combination with such therapies in the future. Because inflammatory responses, even if localized, can affect the immune system throughout the body, investigating the impact of inflammatory responses on surrounding cells and tissues as well as distant locations might be another topic for future research. Elucidating the systematic responses induced by the immune response will contribute to our understanding of the pathogenesis of infectious diseases and autoinflammatory diseases, and developing appropriate treatments.

## Author Contributions

All authors conceptualized the framework of this review article, corrected, read, and finalized the article, and approved the submitted version.

## Conflict of Interest

The authors declare that the research was conducted in the absence of any commercial or financial relationships that could be construed as a potential conflict of interest.
